# Validity of Dietary Assessment Methods When Compared to the Method of Doubly Labeled Water: A Systematic Review in Adults

**DOI:** 10.3389/fendo.2019.00850

**Published:** 2019-12-17

**Authors:** Tracy L. Burrows, Yan Yee Ho, Megan E. Rollo, Clare E. Collins

**Affiliations:** School of Health Sciences, Faculty of Health and Medicine, Priority Research Centre for Physical Activity and Nutrition, University of Newcastle, Callaghan, NSW, Australia

**Keywords:** dietary assessment, doubly labeled water, validation, adults, energy intake

## Abstract

Accuracy in quantifying energy intake (EI) using common dietary assessment methods is crucial for interpreting the relationship between diet and chronic disease. The aim of this systematic review was to evaluate the validity of dietary assessment methods used to estimate the EI of adults in comparison to total energy expenditure (TEE) measured by doubly labeled water (DLW). Articles in English across nine electronic databases, published between 1973 and February 2019 were retrieved. Studies were included if participants were adults (≥18 years) and used the DLW technique to measure TEE compared to self-reported EI. A total of 59 studies were included, with a total of 6,298 free living adults and a mean of 107 participants per study. The majority of studies including 16 studies that included a technology based method reported significant (*P* < 0.05) under-reporting of EI when compared to TEE, with few over-reporting EI. Misreporting was more frequent among females compared to males within recall based dietary assessment methods. The degree of under-reporting was highly variable within studies using the same method, with 24 h recalls having less variation and degree of under-reporting compared to other methods.

## Introduction

The accuracy of measuring food and nutrient intakes using various dietary assessment methods is crucial for interpreting the relationship between development of dietary related chronic diseases, including type 2 diabetes mellitus, cardiovascular disease, and some cancers (1). These chronic diseases contribute significantly to the global burden of disease (2). The validity of dietary assessment methods plays an important role in accurately describing the dietary patterns and nutrient intakes of populations, comparing dietary intakes to recommended dietary guidelines, and following trends in dietary intakes in populations over time (3–5). While self-report measures of EI have received criticism, recommendations have been made to minimize bias when collecting, analyzing, and interpreting dietary data assessed using self-reported methods (6).

The incorporation of technologies to assess dietary intake, including by way of smartphone and the Internet, has facilitated key developments in the collection, analysis and interpretation of dietary intake data (7). This includes reducing costs associated with data collection and analysis, lowering subject and researcher burden and facilitating more timely approaches to data analysis (7). However, the emergence of newer dietary assessment methods with technology assisted components, such as image-based methods and wearable devices (e.g., micro-camera) that incorporate technology for data collection, means that a review of the validity of technology based methods is also timely (8, 9).

A variety of established self-reported dietary assessment methods exist, including 24 h recalls, diet histories, food frequency questionnaires (FFQs) and food records. Many methods are subject to mis-reporting which is often classified as over- or under-reporting (10, 11), with an additional selection bias in terms of the type of people who volunteer to participate in these studies, due to high participant burden (12, 13). Other potential biases within assessment of dietary intake can stem from issues relating to memory, perception and conceptualization of portion sizes, knowledge and confidence with technology—all of which could impact adversely on accuracy of reported EI (14, 15).

Image-based methods require participants to capture digital images of food and beverages pre- and post-consumption with a camera device, and as such are similar to a food record (7). Image-based methods may be susceptible to mis-reporting due to reactivity bias, in that knowing one must take an image of the foods about to be eaten may influence what foods the person chooses to eat on that occasion (9). In addition, measurement using a technology based dietary intake method is dependent and subject to the inherent limitations of technology-based approaches; identification of the food and its components and accounting for intra- and inter-individual variability, and complexities (7) related to whether food is consumed from one's own plate or shared plates (16) and/or consumed with additional condiments.

Measuring the validity of dietary assessment tools requires an objective measure that does not face the same inherent errors found in the dietary assessment tool being assessed. The doubly labeled water (DLW) technique is an objective method of measuring total energy expenditure (TEE), and is considered a reference method for evaluating validity of self-reported EI in relatively weight stable individuals (3, 4, 12). It is also independent of self-reported error (17, 18). An initial DLW dose is determined by standardized equations according to body weight. Following consumption, urine samples are collected over a period of seven to 14 days to account for short-term day-to-day variation in physical activity (19).

A previous review (2001) provides valuable insight that EI is consistently under-reported compared against DLW, with the majority of studies at the time of publication using food records or diaries (17). An additional review by Hill and Davies in 2001 went further to describe characteristics associated with under-reporting which included: (1) Dietary restraint, (2) Socioeconomic status, and (3) Gender (under-reporting more common in women than in men) (20). An additional review by Livingstone and Black (21) detailed additional factors relating to low energy reporters, which included possible cultural influences. However, there have been no reviews in adults since that investigate the misreporting of energy intake. It is within this context that this review aims to evaluate the validity of self-reported dietary assessment methods in estimating the daily EI of adults (≥18 years) in comparison to TEE measured by DLW.

## Materials and Methods

### Search Strategy

Initially searches of online database were conducted in Cochrane, CINAHL, MEDLINE, EMBASE, Scopus, Cumulative Index to Nursing and Allied Health Literature, ProQuest, PubMed and Excerpta Medica Database. Keywords and combinations of keywords used included adult, dietary assessment, food frequency questionnaire, dietary recall, 24 h food recall, diet record, food record, food diary, energy intake, energy expenditure, doubly labeled water, valid^*^, accuracy^*^, precise^*^ and combination of all above-mentioned, see Supplementary Material for example search strategy. Articles retrieved were limited to those published in English-language journals between 1973 and February 2019. The reference lists of articles that met the inclusion criteria were hand searched and key articles identified were used for further searches via the Web of Science database Cited Reference function. Authors were not contacted for any missing information and gray literature was not searched. The protocol for this review was developed and registered with PROSPERO—an international prospective register of systematic reviews, under the registration number CRD42017064545.

### Study Selection

**Figure 1:**
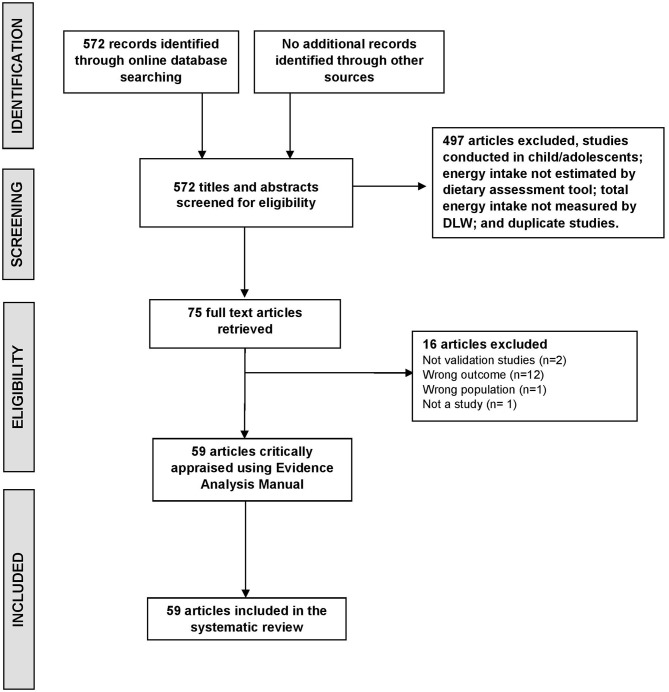
Flow diagram of method for determining studies to be included in this systematic review of evaluating dietary assessment methods against the gold standard doubly labeled water method (DLW).

The flow of studies at each stage of the review is depicted in Figure 1. Following the initial database searches, titles and abstracts were screened to determine which studies required full text retrieval. The full-text articles retrieved were assessed for eligibility using inclusion criteria. The screening was done by two independent reviewers (Y.H and T.B). Articles were identified as relevant if they were studies that aimed to compare dietary intake with TEE, if they included adult participants (aged ≥18 years), if they reported EI measured by a dietary assessment method, if DLW was used to estimate TEE and if the primary purpose of the study was to validate the dietary assessment method. Full articles were retrieved if eligible for inclusion or if eligibility for inclusion was unclear after screening the abstracts. Articles were reviewed by two independent reviewers (YH and TB). Any disagreement between the two reviewers was resolved by discussion with a third independent reviewer (MR).

### Data Extraction and Quality Evaluation

All relevant articles were then independently assessed for quality using the American Dietetic Association quality checklist for primary studies as outlined in the Evidence Analysis Manual (22). A study was rated as ‘positive’ quality if it satisfied a majority of the quality criteria, including four priority criteria pertaining to (1) Selection of study participants, (2) Comparability of study groups, (3) Intervention description and (4) Outcomes. A study was rated as having “neutral” or “negative” quality based on the number of criteria that were met/ not met. No studies were excluded from the review based on quality assessments.

Data relevant to this review were extracted using a standardized tool which was initially piloted using four studies, with minor wording changes made for reviewer clarity. Data were then extracted by two independent reviewers (YH, TB), including study design participant characteristics, dietary assessment methods(s) used, and DLW results. Any discrepancy was resolved via discussion with a third reviewer (MR). Dietary assessment methods were categorized using the National Cancer Institutes of Health Dietary Assessment Primer definitions (23). Dietary assessment methods with technology components were also recorded if any form of communication and/or information technology was used, such as mobile or smartphone, the Internet or sensors collecting image, movement or auditory data. The technology could be utilized in either the collection, analysis or interpretation of the dietary method.

## Results

### Population

The search strategy identified 572 records (Figure 1). After review of full text papers, 59 articles were included and underwent critical appraisal and data extraction. Major reasons for exclusion were: the study did not report dietary validation results (*n* = 12), not a study (*n* = 3) or not conducted in an adult population (*n* = 1). Table 1 summarizes study details including number of participants and anthropometry, dietary assessment methods used and DLW reporting period. Across the 59 included studies there was a total of 6,298 adults. The majority of studies were conducted in free-living settings with one conducted in a military population (78), one in clinical population group with short bowel syndrome (36), one in obese pregnant women (60) and one in wrestlers (71). The mean number of participants per study was 107 (ranging from 6 to 1075) with the age of participants ranging from 18 to 96 years.

**Table 1:** Characteristics of studies identified in a systematic review of the validity of dietary assessment methods used in adults (≥18 years) when compared with the method of doubly labeled water (DLW).

**References**	**Dietary recall method**		***n***	**Sex**	**Age (y)**		**Participants BMI** **Mean ± SD**	**Length of DLW Collection (days)**
	**Method and recording period**	**Technology component (Yes/No)**			**Range**	**Mean ± SD**		
Andersen et al. (24) Norway	FFQ: recording period 1 year	No	17	All female	NR	23.7 ± 2.5	21.8 ± 2.2	10
Arab et al. (25) USA	6 × 24 h MPR via web based platform delivered over 2 weeks and 6 days FFQ (DHQ): recording period 1 year	Yes	233	Females (*n* = 158) Males (*n* = 75)	21–69	Median (IQR) Overall: 33.3 (12.5) White participants (*n* = 118): 29.2 (13.2) Black participants (*n* = 115): 38.3 (11.8)	Median (IQR) Overall: 25.0 (6.1) White participants: 23.6 (4.8) Black participants: 26.9 (6.7)	15
Barnard et al. (26) Australia	DH with food checklist and WFR: recording period 7 days	No	15	Females (*n* = 8) Males (*n* = 7)	22–59	Overall: 36.2 ± 11.7 Female: 37.1 ± 9.6 Male: 35.4 ± 13.1	Overall: 24.9 ± 4.6 Female: 23.8 ± 5.3 Male: 25.9 ± 3.9	14
Bathalon et al. (27) USA	WFR: recording period 7 days, FFQ: reporting period 6 months and 3 × 24 h recall	No	60	All female	NR	Mean ± SEM Restrained eaters (*n* = 34): 60.3 ± 0.6 Unrestrained eaters (*n* = 26): 59.4 ± 0.6	Mean ± SEM Unrestrained eaters: 23.6 ± 0.6 Restrained eaters: 24.8 ± 0.5	15
Beasley et al. (28) USA	Up to 5 × 24 h recall MPR (phone and in person)	No	450	Males (*n* = 174) Females (*n* = 276)	18–74	18–44 (*n* = 172) 45–64 (*n* = 252) 65+ (*n* = 26)	Underweight (BMI <18.5): *n* = 4 Normal (18.5–24.9): *n* = 85 Overweight (25–29.9): *n* = 180 Obese (25–29.9): *n* = 181	12
Black et al. (29) UK	WFR: recording period 16 days over a period of 1 year for female and male participants and 21 days for post-obese participants defined as having lost more than 12.7 kg and maintained weight loss for more than 6 months. Post obese subjects WFR 10–11 days	No	56	Females (*n* = 18) Males (*n* = 27) Post-obese (*n* = 11)	50–87	Female: 57.9 ± 4.6 Male: 67.5 ± 5.03 Post-obese: 35.6 ± 0.10	Female: 25.0 ± 3.9 Male: 25.4 ± 3.6 Post-obese: 23.6 ± 2.8	14–21
Black et al. (30) UK	DH: reporting period 1 year and WFR: recording period 16 days. PETRA system recorded onto cassette Unstructured 24 h recall; reporting period 7 days	No	16	All female	50–65	57.5 ± 4.6	25.1 ± 4.2	14
Blanton et al. (31) USA	2 × 24 h MPR using automated computerized recall. Food Record (FR): recording period 14 days, FFQ: reporting period 1 year. DHQ (FFQ): reporting period 1 month	Yes	20	All female	25–40	30.0 ± 3.9	22.1 ± 1.9	15
Boushey et al. (32) USA	Mobile food record for 7.5 days Select foods provided for study	No	45	Females (*n* = 30) Males (*n* = 15)	21–65	33 ± 12	26 ± 6	8
Champagne et al. (33) USA	WFR: recording period 7 days	No	20	All females	NR	Dietitians (*n* = 10): 36.4 ± 3.8 Non-dietitians (*n* = 10): 33.4 ± 2.0	Dietitians: 23.0 ± 1.1 Non-dietitians: 23.1 ± 1.2	7
Christensen et al. (34) Sweden	Web-based FFQ (normal) 174 items, Web-based FFQ (mini) 126 items and web-based FR: recording period 7 days	No	39	Females (*n* = 31) Males (*n* = 8)	20–63	33.0 ± 12.0	23 ± 3.7	11
Farooqi et al. (35) Sweden	FR: recording period 7 days and DH	No	19	All females	60–80	69.2 ± 6.0	24.5 ± 3.5	14
Fassini et al. (36) Brazil	4 × 24 h recalls (3 weekdays and 1 weekend day) using USDA multi pass method (in person and phone)	No	22	Females (*n* = 12) Males (*n* = 10)	37–65	53 ± 8	Short bowel syndrome (SBS) group: 21.5 ± 3.4 Control group 22.3 ± 2.5	14
Ferriolli et al. (37) Brazil	FFQ	No	19	Females (*n* = 9) Males (*n* = 10)	60–75	Female: 66.5 ± 4.6 Male: 66.2 ± 3.3	Female: 29.3 ± 6.3 Male: 26.8 ± 4.4	10
Freedman et al. (38) USA	2 × FFQ and 2 × 24 h MPR, interviewer administered	No	484 Repeat DLW measures *n* = 25	Females (*n* = 223) Males (*n* = 261)	40–69	NR	NR	14
Gemming et al. (39), New Zealand	3 × 24 h MPR interviewer administered 3 × wearable camera assisted 24 h MPR (MPR+SC)	Yes	40	Females (*n* = 20) Males (*n* = 20)	18–64	Female: 27.1 ± 7.5 Male: 34.8 ± 12.6	Female: 22.3 ± 2.3 Male: 27.1 ± 3.9	15
Hagfors et al. (40) Sweden	DH: Reporting period 1 month	No	9	Females (*n* = 6) Males (*n* = 3)	48–67	Total group. MD: 58.8 ± 9.9 CD: 59.5 ± 8.1	Total group. MD: 28.1 ± 4.4 CD: 26.4 ± 3.2	14
Hise et al. (41) USA	Observer recorded WFR and 14 × 12 recalls to capture snacks outside a controlled cafeteria setting	No	54	Females (*n* = 32) Males (*n* = 22)	NR	Female: 22.1 ± 4.3 Male: 22.7 ± 3.8	Female: 29.5 ± 2.8 Male: 30.3 ± 2.9	14
Howat et al. (42) USA	2 × 24 h recall and FR: recording period 14 days	No	44	All female	18–49	Experimental group (*n* = 18): 33 ± 9.92 Control group (*n* = 26): 34 ± 9.39	BMI range: 16.88 to 64.45 BMI <19: *n* = 8 BMI 19–24: n = 14 BMI 24–27: *n* = 6 BMI >27: *n* = 16	7
Hutchesson et al. (43) Australia	Web-based FR: recording period 9 days	Yes	9	All female	20- 48	34.5 ± 11.3	29.2 ± 1.4	10
Johnson et al. (44) USA	4 × 24 h MPR over 14-day period interviewer administered (2 in person, 2 over the phone)	No	35	All female	19–46	30.2 ± 6.7	28.3 ± 7.4	14
Kaczkowski et al. (45) Canada	Multimedia (cassette and camera) FR: recording period 4 days	Yes	53	All female	50–93	64.9 ± 11.3	24.4 ± 4.0	13
Koebnick et al. (46) Germany	FR: recording period 4 days	Yes	29	Females (*n* = 16) Males (*n* = 13)	19–64	36.8 ± 11.8	23.4 ± 2.7	14
Koehler et al. (47) Germany	FR: recording period 7 days	No	14	All male	NR	30.4 ± 6.2	23.2 ± 1.4	7
Kroke et al. (48) Germany	12 × 24 h recall, computer assisted l and FFQ: 146 item reporting period 1 yea	Yes	28	NR	40–67	Males: 56 ± 7.6 Females: 52 ± 4.7	Males: 26.9 ± 3.7 Females: 26.1 ± 4.65	14
Lins et al. (49) Brazil	3 × 24 h recall and FFQ	No	67	All female	19–45	30.94 ± 5.36	27.7 ± 5.05	14
Lissner et al. (50) USA	2 × 24 h MPR and FFQ all interviewer administered: Reporting period 1 year	No	390	Females (*n* = 179) Males (*n* = 211)	40–69	NR	BMI <25 (females *n* = 75, males *n* = 55) BMI 25–29 (females *n* = 54, males *n* = 99) BMI ≥ 30 (females *n* = 50, males *n* = 57) 80% Caucasian	NR
Livingstone et al. (51) Ireland	WFR: recording period 7 days	No	31	Females (*n* = 15)Males (*n* = 16)	NR	Females 35.5 ± 11.4 Males 31.5 ± 7.2	Female: 24.3 ± 3.1 Male: 25.8 ± 3.3	15
Lof et al. (52) Sweden	3 × 24 h recall via phone	No	37	All female	21–41	29 ± 4.0	23.0 ± 3.0	15
Lopes et al. (53) Brazil	3 × 24 h MPR completed in person and FR—recording period 2 days interviewer administered	No	83	Females (*n* = 50) Males (*n* = 33)	45–60	NR	BMI <25: (females *n* = 15, males *n* = 8) BMI ≥ 25: (females *n* = 35, males *n* = 25)	10
Mahabir et al. (54) USA	FR: recording period 7 days and DHQ (FFQ)	No	65	All female	49–79	59.9 ± 7.5	27.7 ± 5.6	14
Martin et al. (55) USA	Remote Food Photography Method (RFPM): recording period 6 days	Yes	40	Females (*n* = 44) Males (*n* = 6)	18–65	43.0 ± 14.3	31.9 ± 3.7 Caucasian 77%	14
Martin et al. (56) Canada	WFR: recording period 7 days	No	29	All female	37–57	48.7 ± 5.0	23.3 ± 2.5	13
Medin et al. (57) Norway	Web FFQ (1 year reporting period, 279 foods), four 24 h non-consecutive days conducted via telephone using 3 step approach	Yes	29	All female	NR	38.5 ± 10.7	23.8 ± 3.7	14
McClung et al. (58) USA	Hand-held personal digital assistant (PDA): recording period 7 days or written FR: recording period 7 days	Yes	26	Females (*n* = 2) Males (*n* = 24)	NR	23.0 ± 4.0	24.0 ± 2.0 ADF personnel	9
Moshfegh et al. (59) USA	3 × 24 h MPR: 1 completed in person and 2 by phone computer assisted	No	524	Females (*n* = 262) Males (*n* = 262)	30–69	Not reported	21% of sample were obese Non-Hispanic white 77%	14
Most et al. (60) USA	SmartIntake—smart phone application based on the RFPM method. Food images collected for 6–7 days with written records collected if missed image	Yes	23	All female	18–40	± 1.1	36.9 ± 1.3 Obese class I (*n* = 10), obese class II (*n* = 7), obese class III (*n* = 6)	7
Nybacka et al. (61) Sweden	FR: recording period 4 days and FFQ, reporting period previous few months	No	40	Females (*n* = 20) Males (*n* = 20)	50–64	Females: 57.8 ± 4.1 Males:58.6 ± 4.9	Female: 25.7 ± 3.1 Male: 27.3 ± 3.0	14
Okubo et al. (62) Japan	DHQ (FFQ): reporting period 1 month	No	140	Females (*n* = 73) Males (*n* = 67)	20–59	Females: 38.5 ± 10.4 Males: 39.4 ± 11.1	Female: 21.6 ± 2.7 Male: 23.3 ± 2.9	14
Park et al. (63) USA	6 × ASA24, 2 × FFQ (134 items), 2 × unweighed 4DFR (paper based) and 7-day food checklist (32 items)	No	1075 *n* = 704 (DLW)	Females (*n* = 545) Males (*n* = 530)	50–74	Males: 64 Females: 62	Female: BMI 30 to <40 *n* = 32 Males: BMI 30 to <40 *n* = 29	10
Persson et al. (64) Sweden	FR: reporting period 7 days collected by ward ward staff	No	31	Females (n = 18) Males (n = 13)	65–96	86 ± 6	Female: 22.6 ± 3.6 Male:24.2 ± 3.4	22
Pettitt et al. (65) UK	FR: reporting period 14 days FR with wearable micro-camera (FRMC) worn on ear: reporting period 2 days	Yes	6	Females (*n* = 2) Males (*n* = 4)	24–34	28.5 ± 3.39	BMI: 25.3 ± 2.6	14
Pfrimer et al. (66) Brazil	FFQ: reporting period 1 year interview administered and 3 × 24 h MPR	No	41	Females (*n* = 21) Males (*n* = 20)	60–70	Females: 67 ± 3 Males: 68 ± 4	Female: 29 ± 5 Male: 26 ± 4	10
Ptomey et al. (67) USA	Digital photographs for 7 days and 7 × 24 h MPR (DPR)	Yes	91	Females (*n* = 45) Males (*n* = 46)	18–30	Overall: 22.9 ± 3.2 Females: 22.4 ± 3 Males: 23.4 ± 3.4	30.6 ± 4.6 Female: 29.5 ± 4.5 Male: 31.7 ± 4.4 Non Hispanic and white (*n* = 78)	14
Rafamantanantsoa et al. (68) Japan	FR: reporting period 3 days and camera camera (FRC) for 3 days	Yes	44	All male	30–79	51 ± 14	23.3 ± 2.6	14
Rollo et al. (69) Australia	Nutricam Dietary Assessment Method (NuDAM) on mobile phone consisting of primarily an image-voice food record: reporting period 3 days and WFR for 3 days	Yes	10	Females (*n* = 4) Males (*n* = 6)	48–69	61.2 ± 6.9	31 ± 4.5	14
Rothenberg et al. (70) Sweden	DH: interview, reporting period 1 month	No	12	Females (n = 9) Males (n = 3)	NR	73 (SD NR and described as geriatric)	25 ± 2.8	20
Sagayama et al. (71) Japan	Self-reported WFR (written) and visual record using a digital camera	Yes	10	All male	NR	20.4 ± 0.5	Overall: 25.7 ± 1.7 Lightweight wrestlers: 24.5 ± 0.9 Middle weight wrestlers: 27.5 ± 0.4	7
Sawaya et al. (72) USA	WFR: reporting period 7 days. 24 h recall: reporting period 2 days. FFQ × 2 (Willett): reporting period 1 year. FFQ × 2 (Fred Hutchinson Cancer Research (FHCRC/BLOCK)): reporting period 1 year	No	20	All female	NR	Younger women: 25.2 ± 3.5 Older women: 74.0 ± 4.4	Younger women: 20.9 ± 1.9 Older women: 24.1 ± 2.8	7
Scagliusi et al. (73) Brazil	3 × 24 h MPR, FR: recording period 3 days and FFQ: reporting period 1 month	No	65	All female	18–57	33.7 ± 10.8	27.9 ± 6.7 White (n = 43) Black/mulatto (n = 17) Asian/Brazilian (*n* = 5)	10
Schulz et al. (74) USA	10 × 24 h interviewer administered recall and FFQ: reporting period not specified	No	21	Females (*n* = 9) Males (*n* = 12)	NR	Females 31.3 ± 13.0 Males 35.4 ± 13.8	Female: 42.2 ± 12.5 Male: 32.3 ± 9.4 Pima Indians	14
Shook et al. (75) USA	3x interviewer administered 24 h recalls on random non-consecutive days over a 14-days	No	195	46% female	21–35	27.9 ± 3.8	25.8 ± 4.1	14
Subar et al. (5) USA	2 × 24 h MPR and FFQ (DHQ): reporting period 1 year	No	484	Females (*n* = 223) Males (*n* = 261)	40–69	NR	Female: <25.0 (*n* = 86) 25.0–29.9 (*n* = 72) >30.0 (*n* = 65) Male: <25.0 (*n* = 57) 25.0–29.9 (*n* = 127) >30.0 (*n* = 77)	14
Svendsen et al. (76) Norway	WFR: recording period 3 days and FFQ interviewer administered: reporting period 3 months	No	50	Females (*n* = 27) Males (*n* = 23)	24–64	43.2 ± 10.3	Female: 36.6 ± 3.4 Male: 34.6 ± 2.9 All participants with obesity	14
Svensson et al. (77) Sweden	SDQ (FFQ). Reporting period 3 months. FFQ. Reporting period 1 year (Completed by non-pregnant participants only)	No	90	All female	NR	Median (IQR) Overall: 35.7 (3.3) Non-pregnant: 29.2 (6.6) Pregnant: 31.5 (3.8) Median 29.2, IQR 6.6	Individuals with Overweight/obesity (*n* = 31) BMI median (IQR) Non-pregnant women (*n* = 65) 24.7 (8.8) Pregnant women (*n* = 25) 25.2 (3.6)	10
Tanskanen et al. (78) Finland	2x Pre-filled food diary: reporting period 3–4 days. The prefilled food diary included details of food and fluid and composition of foods served in military so were added to the prefilled diary	No	24	All male	19–20	19.6 ± 0.2	24.3 ± 3.8 All Conscripts—compulsory military service	14
Tran et al. (79) USA	2 × 24 h MPR via telephone and 2 × 24 h MPR in person	No	35	All female	19–46	30.2 ± 6.7	28.3 ± 7.4	14
Weber et al. (80) USA	FR reporting period 8 days. Analyzed using 2 different USA nutrient databases: Nutrient Data System (NDS) Nutritionist III (N3)	No	16	All female	18–32	23.9 ± 5.0	Lean women (*n* = 8): 21.4 ± 2.2 Women with obesity (*n* = 8): 32.0 ± 3.5	8
Yuan et al. (81) USA	2 × SFFQ (152 item—paper based), 2 × 7-day DR and 4 × ASA24	No	624	All female	NR	61.4 ± 9.5	26.5 ± 5.4	14

The majority of studies were conducted in the United States of America (*n* = 25) (5, 25, 27, 28, 31–33, 38, 41, 42, 44, 50, 54, 55, 58–60, 63, 67, 72, 74, 75, 79, 80) in adults of Caucasian ethnicity. Ten studies included participants from a range of ethnicities including; African American (*n* = 6) (25, 41, 50, 54, 55, 73), Native American (*n* = 1) (41) Hispanic (*n* = 3) (31, 41, 59), Asian (*n* = 6) (31, 39, 41, 50, 54, 73), Swedish (*n* = 1) (61), Nordic (*n* = 1) (61), Maori (*n* = 1) or unspecified (*n* = 5) (31, 50, 54, 59, 67). The majority of studies included both male and female participants (*n* = 26), with 23 studies having female participants only and four studies with male participants only (47, 68, 71, 78). Two studies did not report the sex of participants (48, 55). The majority of studies (>70%) measured body weight pre- and post-study, 13 studies measured participant body weight at baseline only (25, 28, 29, 33, 34, 37, 49, 51, 53, 66, 67, 70, 77), and body weight was unclear or not reported for five studies (38, 46, 60, 74, 75). The majority of studies reported minor weight change, while the degree of weight change was not statistically significant in 22 studies.

### Quality Appraisal

Forty-three of the 59 studies were evaluated as having a positive study quality, with 16 rated as being of neutral quality (Table 2). The main reasons for being rated as neutral quality were a lack of detail in describing the intervention/therapeutic regimens/exposure factors and/or procedures or comparators (*n* = 9) (25, 35, 38, 46, 49, 50, 54, 64, 71), statistical analyses not adequately described (*n* = 6) (35, 38, 48, 49, 59, 71), possible bias in participant selection (*n* = 5) (38, 64, 71, 74, 79), possible bias due to funding and sponsorship (*n* = 5) (25, 42, 43, 47, 50), conclusion not supported by results or lack of description of limitations (*n* = 3) (27, 54, 74).

**Table 2:** Quality assessment of included studies.

**Study (1st Author, Year)**	**1. Was the research question clearly stated?**	**2. Was the selection of study participants/** **patients free from bias?**	**3. Were study groups comparable?**	**4. Was method of handling withdrawals described?**	**5. Was blinding used to prevent introduction of bias?**	**6. Were intervention/** **therapeutic regimens/** **exposure factor or procedure and any comparison(s) described in detail?**	**7. Were outcomes clearly defined and the measurements valid** **and reliable?**	**8. Was the statistical analysis appropriate?**	**9. Were conclusions supported by results with biases and limitations?**	**10. Is bias due to study's funding or sponsorship unlikely?**	**Overall quality**
Anderson et al. (24)	Y^a^	Y	NA^b^	Y	NA	Y	Y	Y	Y	Y	P^c^
Arab et al. (25)	Y	Y	NA	NA	NA	UC^d^	Y	Y	Y	UC	Neutral
Barnard et al. (26)	Y	Y	NA	NA	NA	Y	Y	Y	Y	Y	P
Bathalon et al. (27)	Y	Y	NA	NA	NA	Y	Y	Y	UC	Y	P
Beasley et al. (28)	Y	Y	Y	Y	NA	N	Y	Y	Y	Y	P
Boushey et al. (32)	Y	Y	Y	Y	NA	Y	Y	Y	Y	Y	P
Black et al. (29)	Y	Y	NA	NA	NA	Y	Y	UC	Y	Y	P
Black et al. (19)	Y	Y	Y	NA	UC	Y	Y	Y	Y	Y	P
Blanton et al. (31)	Y	Y	NA	Y	NA	Y	Y	Y	Y	Y	P
Champagne et al. (33)	Y	Y	Y	NA	NA	Y	Y	Y	Y	Y	P
Christensen et al. (34)	Y	Y	Y	Y	UC	Y	Y	Y	Y	Y	P
Farooqi et al. (35)	Y	Y	NA	NA	NA	UC	Y	Y	Y	Y	Neutral
Fassini et al. (36)	Y	N	N	N	N	Y	Y	Y	Y	Y	Neutral
Ferriolli et al. (37)	Y	Y	NA	NA	NA	Y	Y	Y	Y	Y	P
Freedman et al. (38)	Y	N	NA	NA	NA	UC	Y	UC	Y	Y	Neutral
Gemming et al. (39)	Y	Y	NA	NA	NA	Y	Y	Y	Y	Y	P
Hagfors et al. (40)	Y	Y	Y	Y	UC	Y	Y	Y	Y	Y	P
Hise et al. (41)	Y	Y	NA	NA	NA	Y	Y	Y	Y	Y	P
Howat et al. (42)	Y	Y	Y	Y	NA	Y	Y	Y	Y	UC	P
Hutchesson et al. (43)	Y	Y	NA	Y	NA	Y	Y	Y	Y	UC	P
Johnson et al. (44)	Y	Y	NA	NA	NA	Y	Y	Y	Y	Y	P
Kaczkowski et al. (45)	Y	Y	NA	Y	NA	Y	Y	Y	Y	Y	P
Koebnick et al. (46)	Y	Y	NA	NA	NA	Y	Y	Y	Y	Y	P
Koehler et al. (47)	Y	Y	NA	N	NA	Y	Y	Y	Y	UC	P
Kroke et al. (48)	Y	UC	Y	NA	UC	UC	Y	UC	Y	Y	Neutral
Lins et al. (49)	Y	Y	NA	Y	NA	Y	Y	Y	Y	Y	P
Lissner et al. (50)	Y	Y	NA	Y	NA	UC	Y	Y	Y	UC	Neutral
Livingstone et al. (51)	Y	Y	NA	NA	NA	UC	Y	UC	Y	Y	Neutral
Lof et al. (52)	Y	Y	NA	NA	NA	Y	Y	Y	Y	Y	P
Lopes et al. (53)	Y	Y	NA	Y	NA	Y	Y	Y	Y	Y	P
Mahabir et al. (54)	Y	Y	NA	NA	NA	UC	Y	Y	UC	Y	Neutral
Martin et al. (55)	Y	Y	Y	Y	NA	Y	Y	Y	Y	Y	P
Martin et al. (56)	Y	Y	Y	Y	UC	Y	Y	Y	Y	Y	P
McClung et al. (58)	Y	Y	Y	Y	Y	Y	Y	Y	Y	Y	P
Medin et al. (57)	Y	Y	NA	N	NA	Y	Y	Y	Y	Y	P
Moshfegh et al. (59)	Y	Y	Y	Y	Y	Y	Y	UC	Y	Y	P
Most et al. (60)	Y	N	NA	UC	NA	Y	Y	Y	Y	UC	Neutral
Nybacka et al. (61)	Y	Y	Y	Y	Y	Y	Y	Y	Y	Y	P
Okubo et al. (62)	Y	Y	NA	Y	NA	Y	Y	Y	Y	Y	P
Park et al. (63)	Y	Y	NA	Y	NA	Y	Y	Y	Y	Y	P
Persson et al. (64)	Y	Y	Y	NA	NA	UC	Y	UC	Y	Y	Neutral
Pettitt et al. (65)	Y	UC	NA	NA	NA	Y	Y	Y	Y	Y	Neutral
Pfrimer et al. (66)	Y	Y	NA	NA	NA	Y	Y	Y	Y	Y	P
Ptomey et al. (67)	Y	Y	NA	NA	NA	Y	Y	Y	Y	Y	P
Rafamantanantsoa et al. (68)	Y	Y	NA	NA	NA	Y	Y	Y	Y	Y	P
Rollo et al. (69)	Y	Y	NA	Y	NA	Y	Y	Y	Y	Y	P
Rothernberg et al. (70)	Y	Y	NA	NA	NA	Y	Y	Y	Y	Y	P
Sagayama et al. (71)	Y	N	NA	Y	NA	Y	Y	UC	Y	Y	Neutral
Sawaya et al. (72)	Y	Y	NA	NA	NA	Y	Y	Y	Y	Y	P
Scagliusi et al. (82)	Y	Y	NA	Y	NA	Y	Y	Y	Y	Y	P
Schulz et al. (74)	Y	UC	NA	Y	NA	Y	Y	Y	UC	Y	Neutral
Shook et al. (75)	Y	Y	Na	Y	NA	Y	Y	Y	Y	UC	Neutral
Subar et al. (5)	Y	Y	NA	Y	NA	Y	Y	Y	Y	Y	P
Svendsen et al. (76)	Y	Y	NA	Y	NA	UC	Y	Y	Y	Y	Neutral
Svensson et al. (77)	Y	Y	Y	Y	Y	Y	Y	Y	Y	Y	P
Tanskanen et al. (78)	Y	Y	NA	Y	NA	Y	Y	Y	Y	Y	P
Tran et al. (79)	Y	UC	NA	NA	NA	Y	Y	Y	Y	Y	Neutral
Weber et al. (80)	Y	Y	Y	NA	Y	Y	Y	Y	Y	Y	P
Yuan et al. (75)	Y	Y	NA	Y	NA	Y	Y	Y	Y	Y	P

a Y, Yes;

b NA, Not Applicable;

c P, Positive;

d *UC, Unclear*.

### Study Design

The reporting period for DLW measurement of TEE ranged from 7 to 22 days (Supplementary Material) 24 h. Five studies collected additional saliva samples for DLW purposes (31, 33, 42, 45, 56) and two also collected blood samples (5, 64).

A total of five different dietary assessment methodologies were used across 59 studies. The most commonly used dietary assessment method was a food record (FR) (*n* = 36), 12 of which were weighed food records (WFR) (26, 27, 29, 30, 33, 41, 51, 56, 69, 71, 72, 76). The range of recording days were 2 and 16 days with the majority (*n* = 12) of studies had a reporting period of 7 days. The next most frequently used method were 24 h recalls (*n* = 24) with the multi-pass method (MPR) used in 13 studies with recall days ranging from two to seven. Seven of the MPR studies had a reporting period of 2 days and an additional six studies reported for 3 days. Of the studies that used a 24 h recall approach (*n* = 24), the range was from two (42) to 14 recalls (41). A total of 18 studies clearly described that they used non-consecutive days for recalls (5, 25, 27, 31, 38, 39, 42, 44, 49, 50, 52, 53, 59, 66, 67, 72, 74, 79, 82).

The next most used method was the FFQ (*n* = 21) (5, 24, 25, 27, 31, 34, 37, 38, 48–50, 54, 61, 62, 66, 72–74, 76, 77, 81) with a reporting period ranging from 1 month to 1 year, with the most frequent reporting period being 1 year (*n* = 8) (5, 24, 25, 48, 50, 66, 72, 77). For studies that utilized the diet history method (*n* = 5) (26, 30, 35, 40, 70) the reporting period was 1 month in two studies (40, 70), 1 year in one study (30), and the other two studies did not specify the reporting period (26, 35). One study utilized a short dietary questionnaire (SDQ) with a reporting period of 3 months (77). Twenty seven studies used one method of dietary assessment, 25 studies utilized two dietary methods within the same study and an additional seven studies (27, 30, 31, 63, 72, 73, 81) used at least three dietary assessment methods.

Eighteen studies used a technology component within a dietary assessment method to estimate EI, with food records the most common (*n* = 10) (43, 45, 55, 58, 60, 65, 67–69, 71), followed by 24 h recalls (*n* = 3) (25, 31, 48) and FFQs (*n* = 3) (34, 39, 57). The technology components included a wearable camera (*n* = 4) (39, 65, 68, 71), digital photos, food photography (*n* = 4) (55, 60, 67, 69), computer/web assisted recalls (*n* = 4) (25, 31, 43, 48) and a handheld personal digital assistant (PDA) (*n* = 1) (58). An additional two studies (29, 45) did not use a technology based method as defined in this review but the dietary assessment method was recorded onto a cassette which was then transcribed. Two studies directly compared a traditional dietary assessment method against one with a technology component. Study results are reported in Table 3.

**Table 3:** Results and outcomes of studies included in a systematic review of the validity of dietary assessment methods used in adults (≥18 years) when compared with the method of doubly labeled water (DLW)^A^.

**References**	**Results**	**Significance of results**	**LOA**	**Correlations EI:TEE**	**Individual level**	**Group**	**Overall quality score^∧^**
Andersen et al. (24) Norway	NS difference between mean EI and mean TEE: −229 kcal/d (± 485). Accuracy was not affected by weight or BMI.	Substantial variability in the accuracy of FFQ at the individual level. FFQ can provide a more accurate measure of the mean EI for groups rather than for individuals.	−1,195 to 717 kcal/d	*r* = 0.36, *p* = 0.15	Under-report: 47%; over-report: 12%.	Under-report: 10%	Positive
Arab et al. (25) USA	Difference in mean EI and mean TEE: 223 kcal/d (diet day) and 662 kcal/d (DHQ). Significant difference (*p* < 0.05) between MPR and FFQ for participants who under-report.	Validity of MPR was superior to that of the FFQ. Ethnicity affects EI accuracy: more under-and-over reporting among whites than blacks, regardless of the method.	Not reported	Diet day: *r* = 0.45; DHQ: *r* = 0.33. Correlations improved with each increased day of recall.	Under-report: 34% (White) vs. 25% (Black) by MPR; 19% (White) vs. 9% (Black) by FFQ.	Under-report: 9% by MPR; 27% by FFQ	Neutral
Barnard et al. (26) Australia	Increased misreporting of EI was associated with increased EE but not with age, sex, BMI or body fat. EI significantly different (*p* = 0.005) between sexes for both DH and FR. NS weight change over study period.	Highly active participants or those with variable dietary and exercise habits are more likely to misreport EI.	Not reported	DH: *r* = 0.90; FR: *r* = 0.79	Adequate-report: *n* = 7 (5 males, 2 females).	Under-report: 47% (female) vs. 1% (male) by DH; 41% (female) vs. 18% (male) by FR	Positive
Bathalon et al. (27) USA	EI accuracy affected by dietary assessment method (*p* < 0.05). Reported EI significantly (*p* < 0.05) lower in restrained eaters. Significant weight change in both groups: −33 g/d (Unrestrained eaters) and −28 g/d (restrained eaters).	Under-reporting higher in restrained eaters. Reporting accuracy tended to be higher for WFR than for 24 h recall or FFQ. Assessing dietary hunger and restraint may help to identify subjects likely to under-report dietary intake.	Not reported	24 h recall: *r* = 0.06, *p* = 0.66; FFQ: *r* = 0.06, *p* = 0.66; WFR: *r* = 0.13, *p* = 0.33	Not reported	Under-report: 11% (unrestrained eaters) vs. 19% (restrained eaters) by WFR; 18% (unrestrained eaters) vs. 24% (restrained eaters) by 24 h recall; 23% (unrestrained eaters) vs. 26% (restrained eaters) by FFQ	Positive
Beasley et al. (28) USA	EI was more highly correlated with TEE among true reporters (within 25% of EI) compared to non-concordant reporters.	Usual intake was correlated with estimated intake and more highly correlated in true reporters compared to non- concordant reporters.	Not reported	*r* = 0.79 (true reporters) vs. *r* = 0.54 (non-concordant reporters)	Reported as true and concordant reporters but values not provided	Not reported	Positive
Black et al. (29) UK	Difference in mean EI and EE affected by BMI: 0.73 (post-obese participants) vs. 0.89 (non-obese participants). EI accuracy not affected by sex: 0.89 (women) vs. 0.88 (men).	EI under-reported in both sexes. Greater under-reporting for post-obese participants.	Not reported	*r* = 0.47, *p* < 0.001	Under-report: *n* = 6.	Under-report: 11%	Positive
Black et al. (30) UK	Mean differences were −1.15 (±1.75) MJ/d for weighed records and −0.43 (±2.40) MJ for diet history. EI accuracy not affected by dietary assessment method: 0.89 (WFR) vs. 0.98 (DH). Mean weight change 0.4 kg (±2.2).	EI under-reported using both methods. Better ranking of individuals by WFR.	WR: −0.2 to −2.6 MJ/d; DH: 1.1 to −2.0 MJ/d	WFR: r = 0.48 DH: r = 0.11 FFQ: r = 0.45 24 h recall: r = 0.44 7 days record: r = 0.24	29% were not classified in the same third of the distribution for energy	Under-report: 2% by DH; 11% by WFR	Positive
Blanton et al. (31) USA	NS difference between mean EI and mean TEE for MPR and FR. Under-reporting by 28% for DHQ and FFQ.	MPR is valid for measuring EI at group level. FR is a valid dietary assessment method. FFQ and DHQ underestimates EI compared to DLW.	24 h recall: −775 to 930 kJ/d; 14 days FR: −1,325 to 346 kJ/d; FFQ: −3,713 to 1,367 kJ/d; DHQ: −3,868 to −1,513 kJ/d	MPR: *r* = 0.53, *p* = 0.02 FR: *r* = 0.41, *p* = 0.07 FFQ: *r* = 0.25, *p* = 0.29 DHQ: *r* = 0.15, *p* = 0.53.	Not reported	Under-report: 28% by FFQ and DHQ NS under-reporting for MPR and FR	Positive
Boushey et al. (32) USA	NS difference between mean EI and TEE. Under-reporting of 12% for men and 10% for women. NS weight change over study period.	Image-based mobile FR as accurate as traditional dietary records. Males more likely to under-report than females.	−1,700 to 700 kcal/d	*r* = 0.58 (*p* < 0.01)	852 kcal/d (men) vs. 444 kcal/d (women).	Over-report: 2% of participants	Positive
Champagne et al. (33) USA	NS difference between mean EI and TEE for Dietitians. Non-dietitians significantly (*p* < 0.05) under-reported EI by 429 kcal/d.	Dietitians reported EI more accurately than non-dietitians. Professional experience and interest in WFR may explain increased accuracy in estimating EI.	Reported in graphical form only	Not reported	Not reported	Under-report: 10%	Positive
Christensen et al. (34) Sweden	Significant (*P* < 0.001) difference between mean EI and mean TEE estimated by both FFQs.	EI under-reported when using the FFQs. validity of WFR superior to that of FFQs.	WFR: −5,800 to 2,246 kJ/d; Mini FFQ: −9,200 to 1,092 kJ/d; FFQ: −8,500 to 1,569 kJ/d	Normal FFQ: *r* = 0.42, *p* < 0.01, Mini FFQ: *r* = 0.38, *p* < 0.01	Not reported	Under-report: 30% by normal FFQ; 36% by mini FFQ; 17% by WFR	Positive
Farooqi et al. (35) Sweden	Significant (*P* < 0.001) under-reporting between mean EI and mean TEE using DH (28%) and FR (20%). EI accuracy was affected by BMI for DH (*r* = −0.47) and FR (*r* = −0.50). NS weight change over study period.	Both DH and FR result in under-reporting of EI in COPD participants. Greater under-reporting by DH than FR.	DH: 5,000 to −500 kJ/d; FR: 5,000 to −1,900kJ/d	DH: *r* = −0.05, *p* = 0.85, FD: *r* = 0.19, *p* = 0.45	More women were valid reporters based on the FR than on DH	Under-report: 28% by DH; 20% by FR	Neutral
Fassini et al. (36) Brazil	NS weight change over study period.	EI under-reporting more prevalent in control group, over-reporting more prevalent in clinical SBS group.	SBS group: −10.3 to 3.9 MJ/d Control: −1.3 to 6.9 MJ/d	SBS group: *r*^2^ = 0.64 Control group: *r*^2^ = 0.01	Not reported	Under-report: 2.9 MJ/d (control); Over-report: 3.2 MJ/d (SBS group)	Neutral
Ferriolli et al. (37) Brazil	Under-reporting of EI highly prevalent. Difference in mean EI and TEE: −17.7%.	EI under-reporting highly prevalent in urban-living Brazilians age 60–75.	Not reported	Not reported	Not reported	Under-report: 13% (female) vs. 20% (male)	Positive
Freedman et al. (38) USA	EI under-reported when using FFQ and MPR.	Less under-reporting by MPR than FFQ.	Not reported	Not reported	Not reported	Under-report: 12% (females) vs. 8% (males)	Neutral
Gemming et al. (39) New Zealand	MPR+SC reduced under-reporting by 6% (women) to 8% (men) compared with the MPR alone (*P* < 0.001). The increase in EI was largely from snack foods. NS weight change over study period.	Use of wearable camera significantly reduced under-reporting for both females and males as compared to MPR only.	Not reported	MPR: *r* = 0.68 (men) vs. 0.82 (women); MPR + SC: *r* = 0.61 (men) vs. *r* = 0.81 (women).	Not reported	Under-report: 13% (females) vs. 17% (male) by MPR; 7% (female) vs. 9% (male) by MPRc	Positive
Hagfors et al. (40) Sweden	NS difference between mean EI and TEE in both Mediterranean-type diet group and control. NS weight change over study period.	DH useful for estimating EI and DH not biased by dietary interventions.	Not reported	Not reported	Under-report: *n* = 3	Under-report: 1%	Positive
Hise et al. (41) USA	Mean EI represented 99% (±18%) of TEE. NS difference between EI and TEE for both sexes, however, females slightly under-reported (3%) and males over-reported (3%). NS weight change over study period.	WFR + 24 h recall is a valid method for measuring EI in a group of overweight and obese individuals but caution should be taken when using it on an individual level.	−1,109 to 1,063 kcal/d	*r* = 0.71	Women: 38 to 398%; men: 30 to 44%	Under-report: 3% female. Over-report: 3% for males	Positive
Howat et al. (42) USA	NS difference between EI estimated by FR and MPR, both under-reported compared with TEE. Training made no difference in validity or reliability but help improve portion size estimates. NS weight change over study period.	FR and MPR may reliable methods, however, are likely to under-report EI. Training may help improve portion size estimates.	Not reported	Not reported	Not reported	Under-report: 21.4%	Positive
Hutchesson et al. (43) Australia	Difference between mean EI and TEE: −2,301 kJ/d. NS weight change over study period.	EI under-reported by overweight and obese females when using web-based FR.	−1,267 to 169 kcal/d	Not reported	Under-report: 44%; over-report: *n* = 0.	Under-report: 20%	Positive
Johnson et al. (44) USA	EI misreporting negatively associated with BMI: *r* = −0.36, *p* < 0.05. NS weight change over study period.	EI under-reported when using MPR at group level. Overweight and obese females are more likely to under-report.	Not reported	24 h recall: *r* = 0.22, *p* < 0.20	Under-report: *n* = 12; over-report: *n* = 1; adequate-report: *n* = 22.	Under-report: 17%	Positive
Kaczkowski et al. (45) Canada	TEE was significantly (*p* < 0.01) higher than reported EI in each age group. NS difference in reporting accuracy among age groups. NS weight change over study period.	EI under-reported when using multimedia FR.	Not reported	Not reported	Not reported	Under-report: 24%	Positive
Koebnick et al. (46) Germany	Mean EI was both over-and-under reported compared with TEE (−49 to 34%). Negative association between EI accuracy and BMI: *r* = −0.39, *p* = 0.04. Accuracy was not affected by sex.	EI tends to be UR when using FR. FR is more useful for estimating EI on a group level than an individual level.	3.5 to −6.4 MJ/d	*r* = 0.69, *p* < 0.01	Under-report: 4%; Over-report: 21%	Under-report: −1.7 ± 2.6 MJ/d	Positive
Koehler et al. (47) Germany	NS difference between mean EI and mean TEE. Significant (*p* < 0.01) proportional bias toward under-reporting in those with high EI	FR useful for estimating EI on a group level but not an individual level.	−1,371 to 1,174 kcal/d	*r* = 0.69, *p* < 0.05 (after removal of implausible reporters)	Not reported	Under-report: 98 kcal/d	Positive
Kroke et al. (48) Germany	Difference between mean EI and mean TEE for both methods were strongly and highly significantly correlated *r* = 0.74, *p* < 0.001. EI accuracy affected by BMI: *r* = 0.50, *p* = 0.007. NS weight change over study period.	EI under-reported by both FFQ and 24 h recall. Possible relation between under-reporting and obesity.	−1,673 to 478 kcal/d	FFQ: *r* = 0.48. No *p*-value reported	Not reported	Under-report: 22%	Neutral
Lins et al. (49) Brazil	NS difference between mean EI and mean TEE when using FFQ (*p* = 0.89). Significantly (*p* = 0.03) higher number of under-reporters in FFQ (*n* = 24) than 24 h recall (*n* = 13). Higher % body fat associated with over-reporting in FFQ but not for 24 h recall.	FFQ useful for estimating EI for groups but lack of precision for individuals. FFQ more useful than 24 h recall estimating EI in low-income populations.	24 h recall: 870 to −1,545 kcal/d; FFQ: 1,500 to −1,888 kcal/d.	Not reported	Under-report: 20% by 24 h recall; 36% by FFQ. Over-report: 5% by 24 h recall; 33% by FFQ.	Under-report: 13%	Positive
Lissner et al. (50) USA	In both obese and non-obese men and women, MPR was more accurate in determining EI. However, both methods under-reported.	Validity of MPR tends to be lower in the group with obesity. No significant difference in validity between obese and non-obese groups for FFQ.	Not reported	24 h recall: *r* = 0.39 (non-obese); vs. *r* = 0.16 (obese) (*p* < 0.01) FFQ: *r* = 0.17 (non obese) vs. *r* = 0.08 obese (*p* = 0.23)	Not reported	Under-report: 7% (non-obese males) vs. 16% (males with obesity) by MPR; 8% (non-obese females) vs. 20% (females with obesity) by MPR. 24% (non-obese males) vs. 31% (males with obesity) by FFQ; 25% (non-obese females) vs. 29% (females with obesity) by FFQ.	Neutral
Livingstone et al. (51) Ireland	When split into thirds of EI, the EI ratio of EI: TEE in the upper third was close to 1.0 with [mean (SE) 0.96 ± 0.08 for females and 1.01 ± 0.11 for males (NS)]. Participants in middle and lower thirds of EI significantly under-reported.	Overall, EI was under-reported when using WFR but could be useful for estimating EI in participants with higher EI's.	Not reported	Not reported	19 individuals considered accurate reporters (±2SD)	Under-report: 18% (females) vs. 19% (males)	Neutral
Lof et al. (52) Sweden	Significant correlation between EI:TEE and BMI (*r* = −0.352, *p* < 0.05). NS weight change over study period.	EI under-reported when using 24 h recall in females aged 21–41 y. Females with higher BMI have a higher tendency to under-report EI.	Not reported	Not reported	Under-report: *n* = 18	Under-report: 22%	Positive
Lopes et al. (53) Brazil	Sex affected reporting accuracy for MPR: more females (29%) under-reporting compared with males (6%) (*p* < 0.05). NS difference between both sexes when using FR. NS difference between EI and TEE for males using both methods or by BMI and age.	EI under-reported by both methods. Both methods more useful in estimating EI in males than females.	MPR: −2,204 to 439 kcal/d; FR: −2,043 to 516 kcal/d	Not reported	Under-report: 32% by food record; 20% by 24 h recall.	Under-report: 31% (females) vs. 24% (males)	Positive
Mahabir et al. (54) USA	Females who were overweight tended to under-report EI more than normal weight females.	EI under-reported by both methods. Greater tendency for females who were overweight to under-report than healthy weight females.	FFQ: 700 kcal to −2,800 kcal/d	Not reported	Not reported	Under-report: 37% by FR; 42% by DHQ	Neutral
Martin et al. (55) USA	Customized prompts did not improve accuracy of mean EI compared with mean TEE, under-reporting by 270 kcal ±748 or 8.8%. No relationship to BMI status.	RFPM is a valid method of estimating EI and is not affected by individual's BMI status.	Reported in graphical form only	Not reported	Not reported	Under-report: 34.3%	Positive
Martin et al. (56) Canada	EI accuracy was not affect by BMI status. NS weight change over study period.	EI under-reported when using WFR in healthy middle-aged females.	Not reported	*r* = 0.46, *p* = 0.01	Not reported	Under-report: 20.2%	Positive
Medin et al. (57) Norway	EI underestimated by both Web FFQ and 24 h recall. NS weight change over the DLW period.	Web FFQ should be used cautiously, however, they seem reasonable for estimating macronutrients and most food groups.	Web FFQ: ± 1.96	Web FFQ: *r* = −0.18 24 h recall: *r* = 0.34	*n* = 14 of 29 women were deemed adequate reporters.	Under-report: 6% by Web FFQ; 17% by 24 h recall	Positive
McClung et al. (58) USA	There is a higher tendency to over and under-report using FR. NS weight change over the DLW period.	PDA is a valid method of estimating EI in a group. Both PDA and FR are less useful in estimating EI at an individual level.	−1,472 to 1,394 kcal/d	PDA: *r* = 0.60, *p* < 0.05 FR: *r* = 0.45, *p* > 0.05	Not reported	Under-report: 8% by FR Over-report: 5% by PDA	Positive
Moshfegh et al. (59) USA	Greater under-reporting of EI with higher BMI.	MPR may be useful for estimating EI in normal weight adults but there is a tendency to under-report as BMI increases.	Not reported	*r* = 0.32 (males) vs. 0.25 (females) *P*-values not reported	Under-report: 20%; over-report: 5%	Under-report: 12% (female) vs. 10% (male)	Positive
Most et al. (60) USA	BMI had a significant effect on EI accuracy (*p* = 0.02). African American women reported significantly lower EI compared with white females (*p* = 0.04). Accuracy better when app used on own phone vs. study provided phone.	SmartIntake—a smartphone application significantly underestimates food intake.	Not reported	Not reported	Not reported	Under-report: 36.6%	Neutral
Nybacka et al. (61) Sweden	Accuracy not affected by ethnicity.	EI under-reported by both methods. FR may be more accurate in estimating EI in a group than FFQ.	FR: 3,000 to −7,000 kJ/d FFQ: 5,000 to −9,000 kJ/d	FR: *r* = 0.12 (men) vs. 0.33 (women) FFQ: *r* = 0.17 (men) vs. −0.05 (women).	Under-report: 40% by FR; 57.5% by FFQ. Over-report: 15% by FR; 5% by FFQ.	Under-report: 20% by FR; 18% by FFQ	Positive
Okubo et al. (62) Japan	EI:TEE ratio was significantly (*P* < 0.05) lower for males than females. Significant (*P* < 0.01) mean weight change in males by −23 ± 55 g/d.	EI under-reported when using DHQ (FFQ) for both males and females.	Not reported	Overall: *r* = 0.35 (*P* < 0.001) *r* = 0.34 (men) vs. 0.22 (women).	Under-report: 58% (male) vs. 32% (female). Over-report: 10% (male) vs. 18% (female)	Under-report: 5–6% (females) vs. 9–16% (males)	Positive
Park et al. (63) USA	Average weight change was −0.3 ± 3.7% for men and 0.1 ± 4.4% for women. Under-reporting highest in participants with obesity and highest for FFQ's.	All EI were under-reported when compared to the DLW method. EI from ASA24 were comparable with 4DFR and both provided the best estimates for dietary intakes.	Not reported	Not reported	Under-report: 13- 32% (male) vs. 21–35% (female) by ASA24; 7–24% (male) vs. 15–20% (female) by FR; 32–46% (male) vs. 20–52 (female) by FFQ	Under-report: 15–17% by ASA24s; 18–21% by 4DFRs; 29–34% by FFQs	Positive
Persson et al. (64) Sweden	Mean weight change throughout study period was −0.5 ± 1.9.	FR may be useful for estimating EI in geriatric patients.	Not reported	Total: *r* = 0.81. *r* = 0.78 (women) vs. *r* = 0.80 (men). All *p* < 0.01.	Agreement by tertile only	Over-report: 8%	Neutral
Pettitt et al. (65) UK	Significant (*p* = 0.04) difference between mean EI estimated by FR and FRMC. NS weight change over study period.	FR with camera provides a more accurate estimation of EI than FR, however, both EI was under-reported by both methods.	14 days FR: 750 to −4,900 kJ/d 2 d FR + camera: 7,800 to 0 kJ/d 2d FR + 2d camera: 0 to −1,100 kJ/d	Not reported	Not reported	Under-report: 34% by FR, 30% by FRMC	Neutral
Pfrimer et al. (66) Brazil	Significant difference between mean EI and mean TEE for different body fatness in females but not in males.	EI under-reported for FFQ and MPR. Females had greater tendency to under-report in both methods. Higher body fatness associated with higher rates of under-reporting, especially for females.	MPR: 479.8 to −971.5 kcal/d; FFQ: 1,303.4 to −1,891.3 kcal/d	FFQ *r* = 0.19, *p* = 0.22, 24 h recall: *r* = 0.25, *p* = 0.11	Under-report: 31% by 24 h recall; 4.5% by FFQ	Under-report: 15.2% (female) vs. 7% (male) by FFQ; 27% (female) vs. 14.2% (male) by MPR	Positive
Ptomey et al. (67) USA	NS difference between mean EI and mean TEE. NS weight change over study period.	DP + R may be useful for estimating EI in overweight and obese young adults.	−1,606 to 1,667 kcal/d for females; −1,266 to 1,460 kcal/d for males	Not reported	Within 10% of the TEE in 35% of participants (31% of men vs. 29% of women).	Over-report: 6.8%	Positive
Rafamantanantsoa et al. (68) Japan	NS correlation between the difference between mean EI and mean TEE and BMI only physical activity. NS weight change over study period.	High intensity physical activity and body composition are important predictors of TEE.	−1,069 to 725 kcal/d	Not reported	Not reported	Under-report: 6%	Positive
Rollo et al. (69) Australia	Mean EI:TEE ratio was 0.76 ± 0.2 and 0.76 ± 0.17 for NuDAM and WFR, respectively. NS relationship between both NuDAM and WFR. NS weight change over study period.	EI under-reported by both NuDAM and WFR. Validity of both methods are similar.	Not reported	Not reported	Under-report: NuDAM (*n* = 3) vs. WFR (*n* = 4). Over-report: *n* = 0	Under-report: 24% by NuDAM and WFR.	Positive
Rothenberg et al. (70) Sweden	Mean EI:TEE ratio: 0.88 ± 0.22.	DH appears to underestimate EI by 12%.	Not reported	*r* = 0.27 (*p* > 0.05)	Under-report: *n* = 4 Over-report: *n* = 1	Under-report: 12%	Positive
Sagayama et al. (71) Japan	Significant difference between initial and final body weight at 73.0 ± 7.9 kg vs. 73.2 ± 8.2, respectively.	EI underestimated in light and middle weight wrestlers.	Not reported	Not reported	Not reported	Under-report: 17% (light weight wrestlers) vs. 23% (middle weight wrestlers)	Neutral
Sawaya et al. (72) USA	EI accuracy not affected by BMI, sex and age. NS weight change during study period.	Most accurate method for younger females was 24 h recall and FFQ (Willett) for older females. Although these methods may be suitable for estimating EI at the group level, none are reliable for at individual level.	Not reported	Willet FFQ: *r*^2^ = 0.40, *p* = 0.05; Block FFQ: *r*^2^ = 0.44, *p* = 0.04.	Not reported	Under-report: 19% (younger female) vs. 22% (older female) by WFR. No under/over reporting (younger female) vs. 25% (older female) by 24 h recalls. 384 kcal/d by FFQ (Willet); 679 kcal/d FFQ (FHCRC/BLOCK)	Positive
Scagliusi et al. (73) Brazil	All three methods showed a lack of concordance with TEE: MPR *r^2^* = 0.02; Food record *r^2^* = 0.03; FFQ *r^2^* = 0.16. Obese participant's under-reported more than normal weight participants for MPR and FR, but not FFQ. Ethnicity was associated with reporting accuracy (*p* = 0.01). BMI was negatively correlated with reporting accuracy for MPR.	FFQ had higher rate of misreporting compared to food FR and 24 h recall, which show similar rates of under-reporting. Weight status affects reporting accuracy and should be considered in studies that rely on self-reports of food intake in females.	MPR: −1,919 to 830 kcal/d; FR: −1,844 to 688 kcal/d; FFQ: −2,235 to 958 kcal/d	MPR: *r* = 0.47 (*p* < 0.01) FR: *r* = −0.39; (*p* < 0.01) FFQ: *r* = −0.10; (*p* = 0.42).	Under-report: *n* = 16 by MPR; *n* = 19 by FR; *n* = 35 by FFQ	Under-report: 21% by MPR; 22% by FR and 24% by FFQ	Positive
Schulz et al. (74) USA	There were NS correlations between EI estimates with both methods and measures of body size.	Both FFQs and 24 h recall under-reported but have comparable accuracy in assessing EI in Native American populations.	Not reported	FFQ: *r* = 0.48, *p* = 0.03 24 h recall: *r* = 0.64, *p* = 0.03	Not reported	Under-report: 20% by 24 h recall; 20% by FFQ	Neutral
Shook et al. (75) USA	Participants were divided into tertiles based on BMI by sex. The difference between estimated EI and DLW was 520, 527, and 788 kcal/d for each tertile.	EI underestimated by 24 h recall and estimates less accurate with increasing weight status.	Not reported	*R*^2^: 0.23	Not reported	Under-report: 611 kcal/d	Neutral
Subar et al. (5) USA	Under-reporting tended to increase with BMI and with increased energy intake. EI accuracy was not affected by age. Over total 3 month study period participants gained weight (1.1 kg for men, 0.5 kg for females).	Under-reporting of EI is higher with FFQ compared to MPR. Females under-reported EI to a greater extent than males for both methods.	Not reported	24 h recall: *r* = 0.39 (women), *r* = 0.24 (men) FFQ: *r* = 0.19 (women); *r* = 0.10 (men).	Under-report: 22% (male) vs. 22% (female) by 24 h recall; 50% (male) vs. 49% (female) by FFQ	Under-report: 12 to 14% (male) vs. 16 to 20% (female) by MPR; 31 o 36% (male) vs. 34 to 38% (female) by FFQ	Positive
Svendsen et al. (76) Norway	Accuracy not affected by sex. Mean weight change in all participants 0·1 kg ± 1·0 (range −3.6 to 1.8 kg).	WFR and FFQs UR EI in obese males and females.	Not reported	Not reported	Under-report: 56% by FFQ; 53% by WFR. Over-report: 8% by FFQ; 2% by WFR	Under-report: 14% (male) vs. 21% (female) by FFQ; 28% (male) vs. 31% (female) by WFR	Neutral
Svensson et al. (77) Sweden	SDQ under-reporting was significantly (*p* = 0.02) higher in females with overweight and obesity (43%) vs. normal weight (22%). Significant correlation between SDQ and FFQ EI to TEE difference (*r* = 0.62; *P* < 0.001). Greater under-reporting in those with higher TEE values.	Both SDQ and FFQ under-reported EI in pregnant and non-pregnant females to a similar extent. A short SDQ is as accurate as a more extensive FFQ in estimating EI in females on a group level.	SDQ: Non-pregnant females = −2,003 to 362 kcal/d; Pregnant females = −957 to 2,121 kcal/d	SDQ r = 0.14 FFQ *r* = −0.05 both NS	Not reported	Under-report: 30% (non-pregnant females) vs. 21% (pregnant females)	Positive
Tanskanen et al. (78) Finland	Reported EI of 11.5 ± 3.2 MJ/d was significantly lower than the mean TEE (15.5 ± 1.6 MJ/d): under-reporting of 26 5% (*p* < 0.001).	Pre-filled food diaries under-reported EI in male military personnel undergoing basic training.	Not reported	*r* = 0.44 (no *p*-value)	Not reported	Under-report: 26%	Positive
Tran et al. (79) USA	NS difference between EI as estimated by telephone MPR compared to in-person (*p* = 0.36). NS weight change over the 14 days period 0.2 (−1.6 to 2.8 kg).	Telephone administered MPR have similar under-reporting as in-person recalls in estimating EI	−811 to 969 kcal	Not reported	Not reported	Under-report: 15% (MPR administered via telephone) vs. 18% (MPR administered in person)	Neutral
Weber et al. (80) USA	In both assessments under-reporting was significantly (*p* = 0.03) higher in obese compared to lean females. Difference between mean EI and mean TEE was 4.6 MJ (obese females) and 3.1 MJ (lean females).	Normal weight and obese females under-reported EI, although the magnitude of under-reporting may be influenced by the database used to assess dietary intake for normal weight females.	NDS: −74 to 1,824 kcal/d; N3: −120 to 1,859 kcal/d	Not reported	Not reported	Under-report: 23% (lean females) vs. 39% (females with obesity) by N3; 30% (lean females) vs. 38% (females with obesity) by NDS	Positive
Yuan et al. (81) USA	ASA24s had lower validity than SFFQ2. SFFQ2 had lower validity than one 7DDR. Averaged 7DDRs had the highest validity.	SFFQ2 provided reasonably valid measurements. The ASA24 needs further evaluation for use in large population studies.	Not reported	SFFQ2: *r* = 0.70, 7-day DR: *r* = 0.63 ASA24: *r* = 0.28	Not reported	Under-report: 15% by SFFQ; 21% by 7-day DR, 17% by ASA24	Positive

∧ *Study Quality assessed by American Dietetic Association tool*.

A *Abbreviations included in above table as defined as follows, LOA, limits of agreement; Y, years; SD, standard deviation; DLW, Doubly labeled water; SFFQ, semiquantitative food frequency questionnaire; FFQ, food frequency questionnaire; BMI, body mass index; MPR, multiple-pass 24 h dietary record; SC, Sensecam; DH, diet history; FR, Food record; WFR, weighed food record; SDQ, short dietary questionniare; PDA, personal digital assistant; NUDAM, Nutricam diet assessment method; RFPM, remote food photography method; DR, dietary record; NS, not significant; EI, energy intake; NDS, Nutrient Data System; N3, Nutritionist III; SBS, Short bowel syndrome; DP + R, digital photographs with dietary recalls; FHCRC, Fred Hutchinson Cancer Research Center*.

### Outcomes by Dietary Assessment Category

#### Food Record

Of the studies that reported the accuracy of food records at the group level, the majority of studies (*n* = 19) found significant under-reporting of EI, by 11 to 41% (26, 35, 42, 43, 46, 53, 54, 61, 65, 73, 78, 80) with over-reporting found in only one study by 8% (64). Three studies found no significant difference between absolute EI estimated by food record and TEE measured by DLW (31, 47, 58).

Six studies using food records reported outcomes by sex (26, 29, 41, 53, 76, 83), with three studies (26, 29, 53) reporting no significant difference between sexes while one study each for males (76) and females (83) identified as having a lower degree of misreporting. One study (41) found that females under-reported while males slightly over-reported.

Two additional studies reported a negative correlation (35, 46) between EI reporting accuracy and BMI while no association with BMI was reported in two studies (56, 72). Two studies found that individuals with overweight and obesity were more likely to under-report compared to normal weight individuals (54, 80), although only one study reported this difference to be statistically significant (*p* = 0.032) (80).

##### Food Record with technology component

Technology was applied to the food record method most commonly using a digital camera (*n* = 4) (45, 67, 68, 71), a mobile phone (image based) (*n* = 3) (55, 60, 69), a wearable camera (*n* = 1) (65), the Internet (*n* = 1) (43), and a PDA (*n* = 1) (58). Of the studies that used a digital camera, three studies reported under-reporting of 6, 17, and 24%, respectively (45, 68, 71) while one study found no significant difference between EI and TEE (67). However, those with overweight or obesity were more likely to over-report EI. Image based methods using a smart phone to estimate EI were under-reported compared to DLW between 20 and 37% (54) and in one study where a wearable camera was used in addition to a food record compared with food record alone, the use of the wearable camera reduced level of under-reporting from 34 to 30% (65).

#### 24-Hour Recall

EI was found to be under-reported by 8–30% (5, 25, 38, 39, 44, 50, 53, 59, 66, 73, 79) across seven studies that evaluated EI reporting by sex. Females tended to under-report more than males in all studies (5, 38, 39, 50, 53, 59, 66, 74). Two studies found a relationship between EI reporting accuracy and weight status, with greater EI under-reporting when expressed as a percentage by overweight/obese adults than normal weight adults (50, 73). One study found EI was over-reported in a clinical group of individuals with short bowel syndrome (36).

##### 24 h MPR with technology component

Technology was mostly added to 24 h recalls through use of a web-based system to assist in standardizing the multiple-pass approach (25, 31, 63). In one study, the 24 MPR method was compared with the same method but with the addition of a wearable camera (39). While both methods were found to under-report EI in comparison with DLW, the camera-based method had a lower degree of under-reporting (13 and 7% for females and 17 and 9% for males for the 24 MPR and 24 MPR with camera, respectively) (39). The camera used in this study was a wearable camera worn around the neck with movement, heat, and light sensors.

#### Studies Using Multiple Methods

Seven studies utilized and reported outcomes of EI mis-reporting using three different dietary methods in one study. The combination of dietary assessment methods most often used were a 24 h recall, FFQ and food records (*n* = 5) (27, 31, 63, 72, 73). Three studies reported that under-reporting was lowest for the MPR method (31, 63, 73), while one reported that food record was lowest (72) and one reported that FFQ was lowest (27).

#### Food Frequency Questionnaire

Significant under-reporting of EI was found at the group level in all studies using an FFQ when compared to the DLW method. EI under-reporting ranged from 4.6 to 42% (5, 24, 25, 27, 31, 34, 37, 38, 48, 50, 54, 61, 62, 66, 72–74, 76, 77). One study showed no significant difference between reported EI and TEE on average when using an adapted version of FFQ from a validated FFQ among low income women in Brazil, however, at the individual level significant misreporting remained (49).

Three studies compared the validity of different FFQs (i.e., Block FFQ vs. National Cancer Institute's Diet History Questionnaire (DHQ) (72) and a full vs. brief FFQ i.e., Meal-Q vs. MiniMeal-Q (28, 34, 72, 77). No significant difference in validity was found between the Block FFQ and DHQ, with both having similar, significant EI under-reporting, by ~27% in 20 female adults (72). The other study found significant (*P* < 0.001) under-reporting of 30 and 36% by both Meal-Q and MiniMeal-Q, respectively. The difference between EI estimated by Meal-Q and MiniMeal-Q was found to be significant (*P* < 0.001) (34). In the study by Sawaya et al. (72), both FFQs were also found to under-report EI in young females.

Sex differences in EI for FFQs were reported in seven studies (5, 37, 38, 50, 62, 66, 76). Three studies reported males misreported to a lesser extent when compared to females (50, 66, 76), two studies reported females misreported to a lesser extent (37, 62), while two studies reported similar amounts or no significant differences (5, 38).

One study using an FFQ identified that individuals with obesity under-reported to a greater extent than their non-obese counterparts (50). Another study indicated that the difference between the EI from a FFQ and the DLW method were significantly correlated with BMI (*r* = 0.50) (48). One study used an FFQ, known as the Short Dietary Questionnaire (SDQ), and identified that EI was significantly (*P* < 0.001) under-reported by ~26%, and that females with overweight/obesity under-reported more than normal weight females (77).

#### Diet History

Four out of the five studies found EI was under-reported by 1.3–47% (26, 30, 35, 70). One study found females under-report to a greater extent than males by 47 and 1.3% respectively (26).

## Discussion

The aim of the current review was to evaluate the validity of self-reported dietary assessment methods used to estimate EI of adults in comparison to TEE measured by the DLW method. A total of 59 studies were included, which utilized a number of dietary assessment methods, of which food records were the most commonly used method (*n* = 36). The main finding from the review is that EI was underestimated for the majority of dietary assessment methods, in the range of 11–41% for food records, 1.3–47% for diet histories and 4.6–42% for FFQs. The method with lowest total amount and lowest level of variation was found to be 24 h recalls, with underestimations of EI ranging between of 8–30%. This variation could be attributed to recall bias, length of reporting period and use of visual aids to estimate portion size.

Methods utilizing a technology component are relatively new compared to traditional methods. They are often more appropriate for some population groups when compared to more traditional methods, such as individuals with language barriers (84). They can also help assist in reducing reliance on respondents' memory and with estimating portion size by capturing intakes in real time *via* images and/or on audio recordings (85). The current review included 15 studies that used a technology component, with only two studies making direct comparisons with traditional methods. The Handheld PDA and the Remote Food Photography Method (RFPM), both categorized as food records, were found to have a lower degree of misreporting, however, these technologies were only supported by one study each (55, 58). For many studies in the current review, the technology component was primarily utilized in the collection phase (31, 34, 39, 43), however, it was unclear in many studies. To date, research estimating EI using wearable devices has been limited to small samples sizes, a limited variety of foods and controlled environments (8, 86). Objective measurement of intake in larger sample sizes and free-living individuals is required to determine the performance of technology based methods, including those that utilize sensors or wearable devices (7).

The current review also identified sex-differences in the validity of EI, with females having a greater tendency than males to misreport EI when using MPR (38, 39, 53), diet history (26) and FFQ. However, for food records and FFQ the differences by sex on self-reported EI were inconsistent (37, 76). In study populations of adults with overweight or obesity, under-reporting of EI was identified to a greater degree compared to adults with normal weight when comparing MPR (50, 73), diet history and food record to TEE using DLW. These results could be reflective of a range of reasons including: difficulty to capture dietary intake using the aforementioned methods in this population group such as differences in portion size or frequency of consumption, as well as dieting practices in these individuals, which has been reported previously (87).

In this systematic review, 32 studies used the method of triads (i.e., 2+ measures of diet + DLW) to evaluate the validity of dietary assessment methods (e.g., FFQ, 24h recall, DLW). Nine of these studies used a technology assisted method (25, 31, 39, 48, 57, 65, 67, 69, 71). The method of triads is a statistical approach occasionally used in dietary assessment research (88–90). This method began to be utilized for validation of dietary assessment methods in the twentieth century and involves three separate methods to measure dietary intake. These could include a primary method and a reference method and a biomarker (90). The method assumes the linearity between the three measurements and the true intake and independence between the three measurement errors. There are several limitations and systemic errors known to affect this approach including the occurrence of correlation coefficients >1 or negative coefficients which limits the application (90).

Interestingly, FFQ was the most common method used in the included validation studies (*n* = 12) (5, 25, 38, 48–50, 54, 61, 66, 74, 76, 77). Similar to other methods, FFQs significantly underestimated EI and its reliability is low due to degree of variation in underestimation across studies with under-reporting ranging from 4.6 to 42%. This may be driven by variation within the FFQ method itself, such as length of reporting period and number of foods and beverages on the questionnaire. Despite this, other dietary assessments, including diet history, FR, WFR, 24 h recall, 24 h MPR and SDQ also underestimated EI. Investigating ways to improve accuracy of estimations of EI are needed and technology-based methods may help to better capture portion size and reduce participant burden (84).

The limitations of using self-reported EI from dietary assessment methods have been previously reported (6, 91). This includes the timeframe of DLW measurements do not necessarily overlap with the period of time covering EIs measurement. If the total EI of participants were atypical during DLW measurement period, the degree of the under or overestimation would be greater than usual. It should also be acknowledged that TEE measured by DLW is not always equal or nearly equal to energy intake in non-weight stable individuals (92, 93). True mis-reporting of EI may have occurred in the included studies. A lack of agreement between methods may be the result of reporter bias or reactivity which occurs when individuals change their dietary behavior due to greater awareness of the measurement of their dietary intake. Reactivity may stem from an individual's desire to reduce burden by simplifying the reporting process (e.g., consuming single foods rather than combination foods) or to comply with socially desirable norms (i.e., to appear to have a healthy diet by reporting intake as per recommended in dietary guidelines).

## Conclusion

The majority of dietary assessment methods included in the current review were found to significantly under-estimate EI when compared to TEE measured using the DLW technique. The degree of under-reporting was highly variable across all methods, however, 24 h recalls were associated with a lower degree of mis-reporting and less variation in degree of under-reporting compared to other dietary assessment methods.

## Author Contributions

TB, MR, and CC designed the review. YH completed a large proportion of the work as part of her final year honors project. All authors contributed to all stages of the title screening, data extraction, and critical review of the manuscript.

### Conflict of Interest

The authors declare that the research was conducted in the absence of any commercial or financial relationships that could be construed as a potential conflict of interest.
