# Supplementary material for: Validity of Dietary Assessment Methods When Compared to the Method of Doubly Labeled Water: A Systematic Review in Adults
Source: Front Endocrinol (Lausanne). 2019 Dec 17;10:850. doi: 10.3389/fendo.2019.00850 (PMC6928130; doi:10.3389/fendo.2019.00850)
Supplement: Supplementary file 1 [file Data_Sheet_1.docx]

Supplementary material: example search strategy

Database(s): **Medline**1946 - present

| **#** | **Searches** | **Results** |
| --- | --- | --- |
| 1 | doubl* label* water.mp. | 1306 |
| 2 | dlw.mp. | 408 |
| 3 | 1 or 2 | 1362 |
| 4 | Nutrition Assessment/ or dietary assessment*.mp. | 13438 |
| 5 | food frequency questionnaire*.mp. | 8914 |
| 6 | ffq.mp. | 2434 |
| 7 | dietary recall.mp. | 1776 |
| 8 | 24 hour food recall.mp. | 101 |
| 9 | diet records/ | 4766 |
| 10 | food record*.mp. | 2543 |
| 11 | food diar*.mp. | 1133 |
| 12 | Energy Intake/ | 36290 |
| 13 | energy expenditure.mp. or Energy Metabolism/ | 79571 |
| 14 | 4 or 5 or 6 or 7 or 8 or 9 or 10 or 11 or 12 or 13 | 131990 |
| 15 | "Reproducibility of Results"/ or valid*.mp. | 801900 |
| 16 | accurac*.mp. | 307126 |
| 17 | precis*.mp. | 286161 |
| 18 | 15 or 16 or 17 | 1243876 |
| 19 | exp Adult/ | 6274099 |
| 20 | adult*.tw. | 1003825 |
| 21 | 19 or 20 | 6791504 |
| 22 | 3 and 14 and 18 and 21 | 262 |
| **23** | **limit 27 to (english language and yr="1973 -Current")** | **261** |
