# Supplementary material for: Validity of Dietary Assessment Methods When Compared to the Method of Doubly Labeled Water: A Systematic Review in Adults
Source: Front Endocrinol (Lausanne). 2019 Dec 17;10:850. doi: 10.3389/fendo.2019.00850 (PMC6928130; doi:10.3389/fendo.2019.00850)
Supplement: Supplementary file 2 [file Table_1.docx]

**Supplementary Table 1. Doubly Labelled Water collection and dosage charateristics**

| **Reference** | **Length  of DLW Collection (days)** | **No.  of  samples** | **Dosage** | **Weight collected  pre and  post study?** |
| --- | --- | --- | --- | --- |
|  |  |  |  |  |
| **Andersen et al,  2003, Norway (80)** | 10 | 10 | 0.18g 100% ^2^H and 0.16g 100% ^18^O per kg BW | Yes |
| **Arab et al,  2011, USA (28)** | 15 | 6 | 2g of 10 atom % ^18^O-labeled water and 0.12g of 99.9 atom % deuterium-labelled water per kg BW | Baseline only |
| **Barnard et al, 2002, Australia (77)** | 14 | 3 | 0.05g ^2^H_2_O and 0.13g H_2_^18^O per kg BW | Yes |
| **Bathalon et al,  2000, USA (29)** | 15 | 7 | 0.15g ^2^H_2_^18^O and 0.075g ^2^H_2_O per kg BW | Yes |
| **Beasley et al, 2016, USA (46)** | 12 | 3 | 1.38g of 10% ^18^O, 0.086 of 99.9% deuterium labelled water per kg BW | Baseline only |
| **Black et al,  1997, UK (56)** | 14 - 21 | 14 – 21 | 0.07g ^2^H_2_O and 0.174g H_2_^18^O per kg BW (female and male participants) and 0.046g ^2^H_2_O and 0.174g H_2_^18^O per kg BW (post-obese). | Baseline only |
| **Black et al,  2000, UK (75)** | 14 | 14 | 0.07g ^2^H_2_O and 0.174g H_2_^18^Og per kg BW | Yes |
| **Blanton et al,  2006, USA (30)** | 15 | 15 | 0.12g 99.9% ^2^H_2_O and 2.5g 10% H_2_^18^O per kg BW  Saliva also collected | Yes |
| **Boushey et al, 2017, USA (47)** | 8 | 7 | 1.8g/kg total body water 10% H_2_^18^O and 0.12 g/ kg of 99% ^2^H_2_O | Yes |
| **Champagne et al, 2002, USA (31)** | 7 | 4 | 0.132g H_2_^18^O and 0.108g ^2^H_2_O per kg BW  Saliva also measured | Baseline only |
| **Christensen et al, 2013, Sweden (57)** | 11 | 11 | 44g ^2^H_2_O added to 1L of 10% normalised H_2_^18^O.  Dose:108g (participants < 75kg) 141g (participants ≥ 75kg) | Baseline only |
| **Farooqi et al,  2015, Sweden (66)** | 14 | NR | NR | Yes |
| **Fassini et al, 2018, Brazil (25)** | 14 | 14 | 2g of H_2_^18^O and 0.12g of 99.9% ^2^H_2_O per kg total body water | Yes |
| **Ferriolli et al,  2010, Brazil (58)** | 10 | 2 | 0.15g H_2_^18^O and 0.07g ^2^H_2_O per kg BW | Baseline only |
| **Freedman et al,  2004, USA (32)** | 14 | 2 | NR | NR |
| **Gemming et al,  2015, New Zealand (51)** | 15 | 5 | 0.1g of 99·9% ^2^H_2_O/kg and 2 g of 10% H_2_^18^O per kg total body water | Yes |
| **Hagfors et al,  2005, Sweden (71)** | 14 | 5 | 0.12g ^2^H_2_O and 0.25g H_2_^18^O per kg total body water | Yes |
| **Hise et al, 2002,  USA (33)** | 14 | 14 | 0.10g ^2^H_2_O and 0.15g H_2_^18^O per kg BW | Yes |
| **Howat et al,  1994, USA (34)** | 7 | 2 | 60g to 100g of ^2^H_2_^18^O depending on BW + baseline saliva | Yes |
| **Hutchesson et al, 2013, Australia (68)** | 10 | 10 | 0.083g ^2^H_2_ and 2.083g ^18^O per kg total body water | Yes |
| **Johnson et al, 1998, USA (35)** | 14 | 5 | 0.12g ^2^H_2_O and 0.15g H_2_^18^O per kg total body water | Yes |
| **Kaczkowski et al, 2000, Canada (69)** | 13 | 4 | 2.5g H_2_^18^O and 0.12g D_2_O kg per total body water + saliva | Yes |
| **Koebnick et al,  2005, Germany (65)** | 14 | 14 | 0.07g ^2^H_2_O and 1.74g H_2_^18^O per kg BW | NR |
| **Koehler et al,  2010, Germany (53)** | 7 | 8 | 0.5g H_2_^18^O and 0.3 D_2_O per kg BW | Yes |
| **Kroke et al,  1999, Germany (55)** | 14 | 14 | NR | Yes |
| **Lins et al,  2016, Brazil (59)** | 14 | 7 | 0.12g heavy water and 2.0g H_2_^18^O per kg total body water | Baseline only |
| **Lissner et al,  2007, USA (36)** | NR | 5 | NR | Yes |
| **Livingstone et al, 1990, Ireland (60)** | 15 | 15 | NR | Baseline only |
| **Lof et al,  2004, Sweden (78)** | 15 | 5 | 0.05g ^2^H_2_O and 0.15g H_2_^18^O per kg BW | Yes |
| **Lopes et al,  2016, Brazil (61)** | 10 | 7 | 2g of 10% H_2_^18^O and 0.12g 99.9% ^2^H_2_O per kg BW | Baseline only |
| **Mahabir et al,  2006, USA (37)** | 14 | NR | 0.14g H_2_^18^O and 0.70g ^2^H_2_O per kg BW | Yes |
| **Martin et al,  2012, USA (38)** | 14 | 6 | 1.425g of 10% enriched H_2_^18^O and 0.075g of 99.9% ^2^H_2_O per kg BW | Yes |
| **Martin et al,  1996, Canada (70)** | 13 | 2 | 0.17g H_2_^18^O and 0.07g D_2_Oper kg BW + saliva | Yes |
| **Medin et al, 2017, Norway (83)** | 14 | 9 | 1.2 ml/kg body mass of 109203.1 parts per million (ppm) ^18^O and 47193.7ppm ^2^H | Yes |
| **McClung et al, 2009, USA (39)** | 9 | 6 | Males: 0.07g ^2^H_2_ and 1.2g 10.1% H_2_^18^O per kg BW  Females: 0.07g ^2^H_2_ and 1.1g 10.1% H_2_^18^O per kg BW | Yes |
| **Moshfegh et al,  2008, USA (40)** | 14 | 14 | 0.10g H_2_O and 0.08g H_2_^18^O per kg BW | Yes |
| **Most et al, 2018 USA (26)** | 7 | 5 | 1.25g of 10% H_2_^18^O and 0.10 g ^2^H_2_O per kg BW | Not clear |
| **Nybacka et al, 2016, Sweden (52)** | 14 | 5 | 0.05g 99.9% ^2^H and 0.10g 10% ^18^O per kg BW | Yes |
| **Okubo et al,  2008, Japan (81)** | 14 | 2 | 0.06g ^2^H_2_O and 0.14g H_2_^18^O per kg BW | Yes |
| **Park et al, 2018, USA (48)** | 10 | 7 | 2 g of 10% and 0.12 g of 99.9% deuterium labelled water per kg body water | Yes |
| **Persson et al,  2000, Sweden (67)** | 22 | 9 | ^2^H_2_O and H_2_^18^O at enrichment of 150ppm for ^2^H and ^18^O. Based on total body water | Yes |
| **Pettitt et al,  2016, UK (73)** | 14 | 8 | 55g 10% H_2_^18^O and 9g 99.9% ^2^H_2_O | Yes |
| **Pfrimer et al 2015, Brazil (62)** | 10 | 5 | 0.12g 99% deuterium-labelled water and 2g 10% ^18^O per kg body water | Baseline only |
| **Ptomey et al,  2015, USA (41)** | 14 | 5 | 0.10g ^2^H_2_O and 0.15g H_2_^18^O per kg BW | Baseline only |
| **Rafamantanantsoa et al, 2003,  Japan (54)** | 14 | 5 | 0.12g ^2^H_2_O and 0.25g H_2_^18^O per kg total body water | Yes |
| **Rollo et al, 2015,  Australia (72)** | 14 | 15 | 0.1g 99% ^2^H and 1.25g 10% ^18^O (per kg BW or total body water NR) | Yes |
| **Rothenberg et al, 1998, Sweden (63)** | 20 | 10 | 0.12g ^2^H_2_O and 0.25 g H_2_^18^O per kg body water | Baseline only |
| **Sagayama et al, 2017, Japan (27)** | 7 | 4 | 1.5g/kg 20% H_2_^18^O and 0.12 g/kg 99.9% ^2^H_2_O per kg body water | Told to maintain weight |
| **Sawaya et al,  1996, USA (42)** | 7 | 10 | 0.07g ^2^H_2_O and 0.15g H_2_^18^O  (per kg BW or total body water NR) | Yes |
| **Scagliusi et al,  2008, Brazil (50)** | 10 | 10 | 0.12g 99.9% deuterium-labelled water and 2.0g 10% ^18^O per kg body water | Yes |
| **Schulz et al,  1994, USA (43)** | 14 | 11 | 3.144 g/kg of BW of a solution made of 20 parts of 10.4 atom % H_2_^18^O and 1 part of 99.9 atom% ^2^H_2_O. | NR |
| **Shook et al, 2017, USA (49)** | 14 | 3 | 1.5ml/kg of BW of H_2_O_2_ | NR |
| **Subar et al,  2003, USA (5)** | 14 | 4 + 2 x 24Hr samples  *DLW collected at 2 time points for a sub sample | 0.12g of 99.9 atom % deuterium and 2g of 10 atom % ^18^O per kg BW  Blood sample also collected | Yes |
| **Svendsen et al,  2006, Norway (76)** | 14 | 8 | 0.05g ^2^H and 0.10g ^18^O/kg BW | Yes |
| **Svensson et al,  2014, Sweden (64)** | 10 | 6 | 0.07g ^2^H_2_O and 0.174g H_2_^18^O per kg BW | Baseline only |
| **Tanskanen et al, 2009, Finland (24)** | 14 | 6 | Weighed mixture of ^2^H_2_O (99.9 atom %) and H_2_^18^O (10 atom %), resulting in initial excess total BW enrichment of 150 p.p.m. for deuterium and 300 p.p.m. for oxygen-18. (gram dosing NR) | Yes |
| **Tran et al,  2000, USA (44)** | 14 | 4 | 0.12g ^2^H_2_O and 0.15g H_2_^18^O per kg body mass | Yes |
| **Weber et al,  2001, USA (45)** | 8 | 5 | 0.15g ^2^H and 0.30g ^18^O/kg BW | Yes |
| **Yuan et al, 2018, USA (74)** | 14 | 6 | NR | Yes |
